# Fampridine and quality of life in individuals with multiple sclerosis

**DOI:** 10.1186/s40064-016-2776-2

**Published:** 2016-07-13

**Authors:** Yoshimasa Sagawa, Eloi Magnin, Laura Paillot, Thierry Moulin, Pierre Decavel

**Affiliations:** Laboratoire d’Exploration Fonctionnelle Clinique du Mouvement, University Hospital of Besançon, 25000 Besançon, France; Regional Memory Centre (CMRR), Department of Neurology, University Hospital of Besançon, 25000 Besançon, France; Department of Neurology, University Hospital of Besançon, 25000 Besançon, France; Integrative and Clinical Neurosciences EA481, Bourgogne Franche-Comte University, 25000 Besançon, France

**Keywords:** Multiple sclerosis, Quality of life, Gait, Fampridine

## Abstract

**Background:**

Fampridine improves walking in patients with multiple sclerosis (pwMS). However, little is known about its impact on the quality of life (QoL) of pwMS.

**Objectives:**

This study aimed to evaluate the contribution of fampridine on the QoL of pwMS and to determine if improvements in QoL are best associated with walk respondents.

**Methods:**

Fifty pwMS were included in this study. The PERSEPP scale and the GaitRite system were used to evaluate QoL and gait respectively. QoL was evaluated 7 days before fampridine (Pre1), on the day the fampridine treatment was initiated (Pre2), and 14 and 21 days after fampridine (Post1 and Post2 respectively). Gait was assessed at Pre-1, Pre-2 and Post-1.

**Results:**

For all patients, fampridine had significant effects (*p* = 0.05–10^−4^, *d* = 0.25–0.45) on the *Overall*, *Relationship difficulties*, *Fatigue*, *Time perspective* and *Symptoms* QoL indices and for gait parameters (*p* = 0.05–10^−4^, *d* = 0.17–0.38). Non-respondents scored significant effects (*p* < 0.05–0.01, *d* = 0.32–0.41) for *Overall, Time perspective* and *Symptoms* QoL indices, whereas respondents scored significant effects (*p* < 0.05–0.01, *d* = 0.51–0.8) for *Overall*, *Relationship difficulties*, *Fatigue* and *Symptoms*.

**Conclusion:**

The QoL of pwMS improved after fampridine, suggesting a real benefit in their lives. However, the contributions to the overall QoL index seem different between groups.

## Background

Multiple sclerosis (MS) is a chronic neurological disorder affecting young and middle-aged adults with a female to male ratio of more than 3:1 (Compston and Coles 2008). The cause of MS is unknown although it involves genetic susceptibility and environmental exposure (Compston and Coles 2008). Since there is no known cure for MS, the main goals of treatment is to delay progression of the disease and to improve health-related quality of life (HRQoL) by masking patients’ symptoms (Compston and Coles 2008).

Among several symptoms of MS (e.g., motor and cognitive impairments, optic neuritis, fatigue, pain, urinary dysfunction) (Smith and McDonald 1999), walking disturbance is traditionally one of the most important and have been used as a major criterion to assess the progression of the disease [e.g., the expanded disability status scale (EDSS) developed by Kurtzke (1983)]. Studies have reported that approximately 75 % of individuals with MS experience clinically significant walking disturbances and this applies even to those with mild EDSS scores (from 1 to 3.5) (Bethoux and Bennett 2011; Johansson et al. 2007). Moreover, walking is related to several daily activities, social participation and independence of individuals (Bethoux and Bennett 2011).

Studies have shown that MS symptomatic treatment by fampridine (4-aminopyridine) is associated with improvements in walking and muscle strength (Allart et al. 2015; Rabadi et al. 2013; Hobart et al. 2013; Goodman et al. 2009, 2010), and possibly with cognition (Jensen et al. 2014), vision, fatigue and spasticity (for a review see Jensen et al. 2014). Indeed, fampridine is a potassium channel blocker which reduces the leakage of ionic current through these channels, prolonging repolarization and thus, enhancing action-potential formation in demyelinated axons (Targ and Kocsis 1985). Presumably, by enhancing action-potential formation, more impulses might be conducted in the central nervous system (CNS) and neurological functions could be ameliorated (Jensen et al. 2014; Hayes et al. 2004). In recent phase III studies, Goodman et al. (2009, 2010) investigated the fampridine effect on the walk of individuals with MS during a timed 25-foot walk test (T25FW). Improvement in walking velocity (≈25 % from baseline) was found for 35–43 % of the individuals in the interventional group.

Regarding determining factors related to HRQoL, mobility was given the highest priority by 65 % of individuals with MS (Bethoux and Bennett 2011). Functional disability measured by patients (i.e., using the Incapacity Status Scale) was significantly associated with both physical and mental component scales from a generic HRQoL questionnaire (i.e., short form-36 health survey) (Gavelova et al. 2015). HRQoL has been defined as subjective satisfaction with life (Schwartz and Frohner 2005). This concept, besides what most people intuitively understand, is difficult to define precisely. It seems important as traditionally used measures of medical outcomes do not sufficiently capture the full impact of the medical intervention in the individuals’ lives (Schwartz and Frohner 2005), and this is especially true in the case of chronic and multidimensional diseases such as MS. However, the fampridine effect on other clinical symptoms of MS as well as on HRQoL measured with MS-specific instruments has not been extensively researched, with studies limiting themselves in most cases to gait (Jensen et al. 2014).

The evaluation of the fampridine effect in terms of gait and HRQoL should bring new insights into its direct and indirect role in individuals with MS. Therefore, this study proposed (1) to evaluate the contribution of fampridine in the HRQoL of individuals with MS and (2) to determine if improvements in HRQoL are more important for walk respondents than for those who seem not to respond to fampridine.

## Methods

### Participants

In an open-label study design, 50 patients with MS were included. All patients met the following inclusion criteria: (1) MS diagnosis regarding the modified Mc Donald criteria (Polman et al. 2011); (2) EDSS status between 4.0 and 6.5; and (3) patients able to walk for at least 6 min. The exclusion criteria were: (1) worsening MS symptoms during the previous 60 days; (2) history of epilepsy or epileptic seizures; (3) immunotherapy change in the previous 60 days; (4) beginning anti-spastic treatment in the previous 30 days; (5) initiation of treatment able to decrease fatigue symptoms in the previous 30 days; (6) modification of the rehabilitation program during the study; (7) renal insufficiency (creatinine clearance <80 ml min^−1^ given by the Kockroft-Gault formula); (8) concomitant treatment by organic cation transporter 2 inhibitor; and (9) hypersensitivity to fampridine. Patients’ characteristics are shown in Table 1.

**Table 1:** Means and (SD) of patients’ characteristics at baseline

Patients’ characteristics	All patients (n = 50)	Non-respondent (n = 35)	Respondent (n = 15)	*p*
Age (years)	51.4 (11.7)	49.2 (10.9)	55.5 (12.3)	0.06
Gender (m/f %)	32/68	30/70	35/65	0.45
EDSS	5.2 (1.1)	5 (1.0)	5.8 (1.1)	*0.01*
Height (m)	1.67 (0.09)	1.67 (0.09)	1.66 (0.08)	0.66
Body mass (kg)	75.5 (17.2)	73.1 (16.9)	80.12 (17.4)	0.17
BMI (kg m^−2^)	27 (5.7)	26 (5.7)	28.9 (5.5)	0.09
Disease duration (years)	14.3 (9.3)	13.5 (8.5)	16.2 (11)	0.36
MS type (RR–SP–PP %)	22–48–32	22–49–29	13–47–40	0.22

The italic value correspond statistical significant differences

*EDSS* expanded disability status scale, *BMI* body mass index, *RR* relapsing–remitting, *SP* secondary progressive, *PP* primary progressive

The study was approved by the ethics committee of the University Hospital of Besançon (n. 13/405) and the French National Security Agency of Medicines and Health Products (ANSM 2013-A002305-56). Written informed consent was obtained from all patients.

### HRQoL evaluation

The PERSEPP scale (*PERception de la Sclérose En Plaques et de ses Poussées*) was used to evaluate the HRQoL of patients with MS (Baroin et al. 2013). This scale takes into account several aspects of HRQoL distributed across 66 items (described below) and includes relapse phases.

Each item contains 6 response types according to a Likert scale where “0” was “strongly disagree” and “5” was “strongly agree”. The PERSEPP scale has been validated in the French language and has a good acceptability (non-return rate <10 %), construct validity (Cronbachs’ α > 0.7) and reliability (ICC: 0.72–0.92) (Baroin et al. 2013).

The 10 PERSEPP indices described below were taken into account for this study. First of all, the *Overall* (33 items) and the *Relapse* (15 items) indices were calculated. Five indices from the 5 dimensions which compose the *Overall* index were also calculated: *Social support* (5 items), *Relationship difficulties (work, family and with partner)* (9 items), *Fatigue* (4 items), *State of mind and associated sleep disorders* (7 items) and *Time perspective* (8 items). To finish, 3 indices from the 3 modules proposed by PERSEPP were evaluated: *Coping* (8 items), *Symptoms* (20 items) and *Treatment* (5 items). Each PERSEPP index was transformed to a range of 0–100 with high values indicating an improved level of HRQoL in a particular dimension.

### Gait evaluation

The gait evaluation was carried out in a dedicated room with the GaitRite system (CIR System Inc., USA), an instrumented walkway embedded with pressure sensors sampled at 120 Hz. The active gait recording surface was 6.10 × 0.61 meters. After appropriate instructions and familiarization, patients were asked to walk with standard sports shoes provided by our laboratory at a fast speed condition (Coleman et al. 2012). To produce a representative gait pattern, a minimum of 10 gait cycles were recorded for each patient. Spatiotemporal gait parameters determined by averaging values across these gait cycles were gait velocity (m s^−1^), cadence (steps min^−1^), step length (m), step time (s), step width (m), stance time (% of gait cycle) and double support time (% of gait cycle).

### Procedures

This study was realized in the Laboratory of Clinical Functional Exploration of Movement at the University Hospital of Besançon. HRQoL was assessed at 4 intervals: 7 days before fampridine treatment initiation (Pre 1); on the day, but before the fampridine treatment initiation (Pre 2); and 14 and 21 days after fampridine treatment initiation, respectively Post 1 and Post 2.

Gait was assessed 3 times at Pre 1, Pre 2 and Post 1. Indeed, fampridine was prescribed according to the guidelines issued by the French National Security Agency of Medicines and Health Products (ANSM) at the dose of 10 mg twice a day. Fampridine is indicated for the improvement of walking in MS patients with a walking disability (EDSS between 4 and 7). According to official ANSM guidelines, the prescription is initially limited to 2 weeks of therapy, at which point a new assessment is performed by the medical practitioner to evaluate the clinical benefits. If no improvement is observed, fampridine should be discontinued.

According to the gait velocity evolution (%) between Pre 1 and Post 1 (i.e., before 14 days after fampridine treatment as recommended by the ANSM), patients were then classified into two groups: *respondents* whose gait velocity improved (in the fast condition) by over 25 % related to the baseline and *non*-*respondents* whose gait velocity did not improve by over 25 %. This conservative threshold corresponds to the average improvement that was maintained throughout the treatment period in the Goodman et al. (2009) study.

### Statistical analysis

Data management and analyses were performed using Statistica version 10 (StatSoft., USA). The results were expressed as mean and standard deviation (SD). 1-way analysis of variance for repeated measurements was used to analyze HRQoL and gait evaluations of all patients after checked normality and homogeneity of variances.

To compare respondent and non-respondent groups (between-factor) during the evaluations (within-factor) in terms of HRQoL and gait, a 2-way analysis of variance was performed. Only the main effects (i.e. the group effect and the fampridine effect) were considered in this study. Tukey post hoc tests were conducted when significant effects existed. This test took into consideration the correction necessary for our 4 evaluations (3 evaluations for the gait) and thus, the level of significance was preserved at 0.05 for all analyses. In addition, effect sizes were calculated to evaluate if differences observed for HRQoL and gait parameters corresponded to important clinical effects (Cohen 1988). The effect size (*d*) is defined as the mean unadjusted difference divided by the pooled standard deviation of the corresponding mean scores. Effect sizes of 0.2, 0.5 and 0.8 were regarded as *small*, *medium* and *large* degrees of differences, respectively.

## Results

### All participants

Table 1 shows the participants’ characteristics as well as those categorized as non-respondent (n = 35, 70 %) and respondent groups (n = 15, 30 %) according to the Goodman et al. (2009) gait velocity threshold. No differences were found between the groups’ characteristics except for the EDSS score. The respondent group had a more important EDSS score than the non-respondent group [EDSS mean (SD): respondent 5.8 (1.5) vs. non-respondent 5 (Compston and Coles 2008); *p* = 0.01, *d* = 0.8].

Regarding the results of all patients for the HRQoL indices, fampridine was found to have significant *medium* effects (*p* = 0.05–10^−4^, *d* = 0.25–0.45) for the following HRQoL indices: *Overall*, *Relationship difficulties*, *Fatigue*, *Time perspective* and *Symptoms* (Table 2). No significant differences were found between the two conditions before (Pre 1 vs. Pre 2) and after fampridine (Post 1 vs. Post 2), except for the *Treatment* index (*p* = 0.001, *d* = 0.5) which was more important at Post 2 than at Post 1 (Table 2).

**Table 2:** Mean and (SD) of the different indices of the PERSEPP HRQoL questionnaire for all patients before (Pre 1 and Pre 2) and after (Post 1 and Post 2) fampridine

PERSEPP (%) (items)	All patients (n = 50)			
	Pre 1	Pre 2	Post 1	Post 2
Overall (33)	62.4 (16.4)	62.5 (16.9)	66.3 (17.6)	68.9 (18.4)*
Social support (5)	87.2 (14.2)	86.5 (16.4)	87.5 (16.7)	88.2 (17)
Relationship dif. (9)	52.2 (22.8)	54.7 (24.1)	59 (24.5)	61.9 (23.2)**
Fatigue (4)	43.5 (30.9)	44 (29.2)	*52.4* (32.8)***	52.8 (34.7)***
State of mind and sleep disorder (7)	63.2 (24.8)	64.3 (26.9)	67.5 (26.6)	69.5 (27.2)
Time perspective (8)	59.2 (22)	57.5 (21.8)	62.4 (24.5)	*66.6 (23.5)* ^+^
Relapse (15) (n = 29)	48.4 (24.4)	50.6 (23)	53.4 (24)	55.7 (28.2)
*Modules*				
Coping (8)	58.7 (19.1)	58.1 (19.)	59.6 (21)	59.5 (20.6)
Symptoms (20)	54.6 (17.9)	55.1 (18.6)	*59.5 (17.7)* ^†^	*63 (18.8)* ^†^
Treatment (5) (n = 26)	86.5 (15.7)	78.6 (25)	80.9 (18.1)	*88 (13.8)* ^¥^

The italic values correspond statistical significant differences

* *p* 10^−4^ Pre 1 and Pre 2 versus Post 2

** *p* < 0.01 Pre 1 versus Post 2

*** *p* < 0.05 Pre 1 and Pre 2 versus Post1 and Post 2

^+^ *p* < 0.01 Pre 1 and Pre 2 versus Post 2

^†^ *p* < 0.05 Pre 1 and Pre 2 versus Post 1, and *p*: 10^−4^ Pre 1 and Pre 2 versus Post 2

^¥^ *p*: < 10^−3^ Post 1 and Post 2

In terms of spatiotemporal gait parameters, considering all patients, significant *small* to *medium* effects (*p* = 0.05–10^−4^, *d* = 0.17–0.38) were found after fampridine (Table 3). For all patients, gait improvement from baseline was comprised between 2.6 % (stance time) and 14 % (velocity).

**Table 3:** Mean and (SD) of spatiotemporal gait parameters for all patients before (Pre 1 and Pre 2 and after (Post 1) fampridine

Spatiotemporal gait parameters	All patients (n = 50)		
	Pre 1	Pre 2	Post 1
Velocity (m s^−1^)	1.05 (0.49)	1.08 (0.51)	1.2 (0.48)**
Cadence (steps min^−1^)	105.18 (28.74)	106.57 (28.98)	112.79 (24.9)**
Step length (m)	0.57 (0.17)	0.58 (0.18)	0.62 (0.16)**
Step time (s)	0.63 (0.25)	0.62 (0.22)	0.56 (0.16)**
Step width (m)	0.13 (0.05)	0.13 (0.05)	0.12 (0.04)*
Stance time (% GC)	66.03 (5.39)	65.83 (5.6)	*64.13 (4.51)* ^£^
Double supp. time (% GC)	32.53 (10.84)	31.91 (11.27)	*29.06 (8.23)* ^£^

The italic values correspond statistical significant differences

* *p* < 0.05 Pre 1 and Pre 2 versus Post 1

** *p* 10^−3^ Pre 1 and Pre 2 versus Post 1

^£^ *p* 10^−4^ Pre 1 and Pre 2 versus Post 1

### Non-respondents and respondents

Table 4 shows the same spatiotemporal gait parameters presented above but categorized as non-respondent and respondent groups. For the non-respondent group, when comparing pre- and post-fampridine treatment, significant *small* effects (*p* < 0.05, *d* = 0.12–0.22) were found for step length, stance time and double support time without an improvement in gait velocity. These gait improvements were comprised between 1.2 and 3.6 % from baseline. For the respondent groups, except for step width, all parameters presented significant *medium* to *large* effects (*p* = 10^−3^–10^−4^, *d* = 0.45–0.9). In terms of gait velocity, improvements after fampridine ranged between 31 % (Pre 2 vs. Post 1) to 56 % (Pre 1 vs. Post 1) from baseline values. The respondent group also presented significant *small* differences (*p* = 0.05–10^−3^, *d* = 0.03–0.06) comparing the two pre-intervention conditions for gait velocity, cadence and step length. This differences, comprising between 5.6 and 19.2 %, were however lower than those found after fampridine treatment (Table 4).

**Table 4:** Mean and (SD) of the spatiotemporal gait parameters for the non-respondent and respondent groups at Pre 1, Pre 2 and Post 1

Spatiotemporal gait parameters	Non-respondent (n = 35)			Respondent (n = 15)		
	Pre 1	Pre 2	Post 1	Pre 1	Pre 2	Post 1
Velocity (m s^−1^)	1.21 (0.44)	1.2 (0.48)	1.27 (0.46)	0.66 (0.36)	0.78 (0.48)*	*1.03 (0.51)* ^£^
Cadence (steps min^−1^)	115.11 (23.03)	113.29 (25.98)	116.35 (23.83)	82.03 (27.97)	90.89 (30.4)**	*104.48 (26.18)* ^£^
Step length (m)	0.62 (0.15)	0.62 (0.16)	*0.64 (0.16)* ^†^	0.46 (0.15)	0.49 (0.18)**	*0.57 (0.16)* ^£^
Step time (s)	0.55 (0.14)	0.57 (0.17)	0.54 (0.14)	0.83 (0.32)	0.75 (0.28)	0.62 (0.18)***
Step width (m)	0.13 (0.05)	0.13 (0.04)	0.12 (0.04)	0.14 (0.07)	0.14 (0.07)	0.12 (0.06)
Stance time (% GC)	64.32 (4.17)	64.58 (4.83)	*63.55 (4.6)* ^†^	70.01 (5.94)	68.76 (6.34)	65.46 (4.12)***
Double supp. time (% GC)	29.1 (8.22)	29.4 (9.66)	*28.12 (8.12)* ^†^	40.53 (12.23)	37.76 (12.86)	31.25 (8.34)***

The italic values correspond statistical significant differences

^†^ *p* < 0.05 Pre 1 versus Post 1

* *p* < 0.05 Pre 1 versus Pre 2

** *p* < 0.01 Pre 1 versus Pre 2

*** *p* 10^−3^ Pre 1 and Pre 2 versus Post 1

^£^ *p* 10^−4^ Pre 1 and Pre 2 versus Post 1

To finish, the analysis of HRQoL for non-respondent and respondent groups found that the non-respondent group scored significant *medium* effects after fampridine (*p* < 0.05–0.01, *d* = 0.32–0.41) for *Overall, Time perspective* and *Symptoms* indices (Table 5). Differences were comprised between 2 and 9 percentage points. The respondent group scored significant *medium* to *large* effects after fampridine (*p* < 0.05–0.01, *d* = 0.51–0.8) for the indices: *Overall*, *Relationship difficulties*, *Fatigue* and *Symptoms.* Differences were comprised between 4 and 22 percentage points.

**Table 5:** Mean and (SD) of the different indices of the PERSEPP HRQoL questionnaire for the non-respondent and respondent groups at Pre 1, Pre 2 and Post 1

PERSEPP (%) (items)	Non-respondent				Respondent			
	Pre 1	Pre 2	Post 1	Post 2	Pre 1	Pre 2	Post 1	Post 2
Overall (33)	63.1 (17.1)	62.5 (16.6)	66.3 (17.6)	68.2 (18.6)*	60.9 (15.1)	62.4 (18.2)	66.4 (18.3)	*70.6 (18.5)* ^**+**^
Social support (5)	85.5 (15.3)	85.5 (12.7)	87.7 (12.4)	87.9 (12.9)	91.2 (10.7)	88.8 (23.3)	87.2 (24.6)	89.1 (24.6)
Relationship dif. (9)	54.8 (23.8)	55.5 (24.4)	62.1 (23.1)	60.9 (22.8)	46.4 (20)	53.1 (23.9)	51.9 (26.7)	*64.1 (25)* ^†^
Fatigue (4)	43 (30.6)	43.6 (28.7)	48.2 (32.4)	47.6 (34.1)	44.6 (32.7)	45 (31.7)	*63.1 (32.7)* ^‡^	*66.2 (34)* ^‡^
State of mind and sleep disor. (7)	64.9 (24.8)	64 (28.8)	65.5 (27.7)	67.8 (28.8)	59.2 (25)	65.1 (22.7)	72.3 (24)	73.5 (23.5)
Time perspective (8)	59.7 (21.7)	58.1 (21.6)	62 (25.3)	66.7 (24.6)*	58 (23.3)	56 (23.1)	63.5 (23.3)	66.4 (21.3)
Relapse (15)	50.9 (26.1)	53.5 (23.9)	56.5 (25.8)	56.9 (30.5)	42 (19.2)	42.1 (19.9)	45.3 (16.7)	52.7 (22.4)
*Modules*								
Coping (8)	59.1 (20.5)	58.8 (20.5)	59.7 (20.8)	59.2 (20.6)	57.8 (15.8)	56.7 (17.2)	59.4 (22.2)	60 (21.2)
Symptoms (20)	56.2 (19)	56.8 (18.9)	*58.9 (19.3)* **	*64.1 (19.2)* **	50.8 (14.9)	51 (17.8)	*61 (14.1)* ^‡^	*60.1 (18.2)* ^‡^
Treatment (5)	86.4 (15.5)	80.8 (21.3)	79.8 (18.9)	87.2 (14.7)***	87 (17.6)	71.3 (36.4)	84 (16.5)	89.1 (11.9)

The italic values correspond statistical significant differences

* *p* < 0.05 Pre 2 versus Post 2

** *p* < 0.01 Pre 1 and Pre 2 versus Pre 1 and Post 2

*** *p* < 0.01 Post 1 versus Post 2

^+^ *p* 0.01 Pre 1 versus Post 2

^†^ *p* < 0.05 Pre 1 versus Post 2

^‡^ *p* < 0.05 Pre 1 and Pre 2 versus Post 1 and Post 2

As expected, the non-respondent and respondent groups were significantly different for all spatiotemporal gait parameters (*p* < 0.01) independent of evaluations (within-factor), except for step width. However, significant differences between the non-respondent and respondent groups were neither found for HRQoL indices nor for the progression (i.e., percentage of mean improvement between Pre 1 and Post 2) of the *Overall* index.

## Discussion

Even if walking disorders are considered the main symptoms of patients with MS, it is well recognized that they do not reflect all the facets that patients consider important in their life. Fatigue, relationship difficulties or sleep disorders are many other examples of a person’s experience with MS. These symptoms could be influenced by walking disorders which in turn could be treated by fampridine (as per the ANSM guidelines) and thus assessed by specific HRQoL and spatiotemporal gait parameters.

Therefore, the aim of this study was to explore the short-term effects of fampridine in the HRQoL of patients with MS and then to determine if the so-called walking respondents seem to better benefit from fampridine treatment in terms of HRQoL. First of all, our results pointed out that fampridine can improve HRQoL in patients with MS as well as their gait. Secondly, contrary to what we could expect, HRQoL improved in both non-respondent and respondent groups. However, for the same rate of overall improvement in HRQoL in both groups, the HRQoL sub-indices that showed significant improvement after treatment were different between the two. This suggests that, as fampridine acts primarily on neurological function, benefits could be perceived differently in groups with different motor impairments.

Previous studies suggest that, based exclusively on walking velocity, only some patients respond with clear clinical benefits to fampridine treatment (Goodman et al. 2009, 2010; Jensen et al. 2014). Using the same protocol and parameter (i.e., walking velocity at fast condition), our results corroborate with these studies where 30 % of patients with MS surpass 25 % of their baseline gait velocity. This result suggests the representativeness of our sample.

Considering that an improvement in walking or functioning capacities may encompass several objectives (e.g., velocity, endurance, dexterity, comfort), recent studies took into account other tests related to MS symptoms (e.g., gait, arm function, fatigue) and found that fampridine may impact on capacities beyond walking function (Allart et al. 2015; Pavsic et al. 2015). Allart et al. (2015) found that 74 % of patients with MS could potentially benefit in some way from fampridine. This was particularly true for fast walkers who completed the T25FW in less than 8 s.

From the perspectives of gait and HRQoL, even after classifying patients into non-respondent and respondent groups, we found an improvement. However, *large* effect sizes for different spatiotemporal gait parameters and HRQoL indices were found for the respondent group. Furthermore, the respondent group surpassed the threshold of Minimally Important Clinical Difference (MICD) reported by Coleman et al. (2012) (i.e., for walking velocity *d* = 0.49). In spite of this fact, the non-respondent group was nearly twice as fast as the respondent group and should not be selected for fampridine treatment. As suggested by a previous study (Allart et al. 2015), *fast* walkers seem to benefit less from fampridine based on walking velocity as a unique point of view. It is important to bear in mind that, even in this non-respondent group, gait velocity was still impaired when compared with normative values. Indeed, the walking velocity assessed in the fast condition in this study for the non-respondent group corresponds to the velocity of a group of women (n = 5013) aged 10 years more and at their self-selected velocity as reported in the Bohannon and Andrews meta-analysis (Bohannon 2006).

Taking into consideration HRQoL, significant *medium* effects were found for all patients with MS. Both groups of non-respondents and respondents presented quite similar values at baseline and after fampridine for all indices (no significant difference between groups). However, the contribution of sub-indices to the *Overall* index seems different between groups. Indices with a significant difference in the respondent group were *Fatigue* and *Relationship difficulties*. These indices seem more related to physical components (see the items of PERSEPP Baroin et al. 2013), suggesting an association with gait improvement on the basis of spatiotemporal parameters. In more detail, the *Fatigue* index is related to tiredness levels and the rest time of a patient. The respondent group showed the most improvement for this index and scored 18–22 percentage points more than the baseline, unlike the non-respondent group which still scored the same value as the baseline. It is probable that fampridine has an important role in fatigue as previously suggested in a study which demonstrated a decrease in general fatigue intensity after fampridine (Allart et al. 2015). The second index, *Relationship difficulties*, concerns more specifically the interactions with others (work, family, and partner/spouse). Improvement in walking velocity (even if it still very low in the respondent group) is one of the best predictors of a person’s independence (Fritz and Lusardi 2009) and related to fall risk (Hars et al. 2013; Cattaneo et al. 2002). This improvement is noted at Post 2 (21 days after fampridine) and it is only after some time with gait improvement that patients are able to observe a change in their social lives.

The non-respondent group in turn showed improvement in the *Time perspective* index. This index corresponds to a view which integrates the totality of the individual’s perspective of his past, present and future at any given time (Zimbardo and Boyd 1999). *Time perspective* seems associated with cognitive functioning (Ernst et al. 2014) or depressive tendencies (Baroin et al. 2013), which may both be modulated by fampridine.

To finish, it seems that there is no convincing clinical profile of patients’ responsiveness to fampridine with our study. The EDSS was the only clinical factor which was significantly different between the non-respondent and respondent groups. The only difference was that individuals in the non-respondent group were categorized as “*Disability precludes full daily activities*” whereas those in the respondent group were categorized as “*Assistance required to walk*”. This difference was not found in previous studies which did not identify any predictive factors for effectiveness (Allart et al. 2015; Goodman et al. 2009, 2010) in spite of the EDSS mean values which had in all cases a worse value in the respondent group.

This study could contribute to knowledge of fampridine’s subjective global effect (i.e., physical, emotional, mental, roles and social functioning) in patients with MS. For this purpose, a disease-specific HRQoL questionnaire with a good reliability was employed. This specific questionnaire should have the advantage of focusing on particular health problems related to MS and might be more sensitive to the detection and quantification of changes related to the disease.

However, this prospective non-randomized study presents several limitations. First of all, the absence of a placebo group might have overestimated the fampridine effect in HRQoL. However, in phase III fampridine clinical trials, the percentage of patients who met the responder criterion in the placebo group was around 9 % in motor and cognitive outcomes suggesting a weak placebo effect (Goodman et al. 2010; Jensen et al. 2014). Secondly, despite the effort to use a specific validated HRQoL questionnaire, at the moment there is no threshold that we could consider as MICD for the PERSEPP. This should be suitable for the non-respondent group whose *medium* effect sizes were found. Third, in spite of the importance of the HRQoL, it is important to be aware of the bias related with self-report questionnaires, which can be influenced by memory deficits or the desire to please the health-care provider. To finish, as with the majority of studies, the fampridine effect was assessed during a short distance test (i.e., the T25FW found for several studies). Although its applicability in clinical practice, this gait test represents neither the range of everyday gait activities (e.g., walking long distances or up and down stairs) nor the participations that result from the walking activity (e.g., going for a walk, shopping, social activities). A recent European multicenter study found that long walking tests were more appropriate than short walking ones in detecting clinically meaningful improvement (Baert et al. 2014). The choice of a gait tests may influence the responder group allocation. The encouraging results of the fampridine on HRQoL should be confirmed by a larger randomized double blind placebo controlled trials.

In conclusion, our study showed that patients with MS improved their HRQoL after fampridine, suggesting a real benefit in their lives. Both non-respondent and respondent groups improved their HRQoL although important effects were seen only in the respondent group. To finish, the contributions to overall HRQoL improvement seem different after fampridine between the non-respondent and respondent groups. The *Fatigue* and *Relationship difficulties* indices contributed significantly to overall improvement for HRQoL in the respondent group whereas, in the non-respondent group, it was *Time perspective* that seemed to better contribute to overall improvement in HRQoL.

## Abbreviations

ANSM: Agency of Medicines and Health Products (ANSM)
CNS: central nervous system
EDSS: expanded disability status scale
HRQoL: health-related quality of life
MS: multiple sclerosis
PERSEPP: *PERception de la Sclérose En Plaques et de ses Poussées*
pwMS: patients with multiple sclerosis
QoL: quality of life
SD: standard deviation
T25FW: timed 25-foot walk test
